# Editorial for the Special Issue “Cadmium and Trace Elements Toxicity”

**DOI:** 10.3390/toxics13121056

**Published:** 2025-12-05

**Authors:** Roberto Madeddu

**Affiliations:** Department of Biomedical Sciences, University of Sassari, 07100 Sassari, Italy; rmadeddu@uniss.it

## 1. Introduction

Cadmium (Cd) and other trace elements represent a significant global environmental and public health concern due to their persistence, bioaccumulation potential, and widespread distribution in ecosystems [1,2,3,4,5]. These toxic metals enter the environment through both natural processes and anthropogenic activities, including industrial emissions, mining operations, agricultural practices, and the extensive use of phosphate fertilizers [6,7,8,9,10]. The non-biodegradable nature of cadmium, coupled with its high soil-to-plant transfer rates, makes it particularly hazardous to human health and environmental integrity [11,12,13].

The toxicological implications of cadmium and trace element exposure are multifaceted, affecting virtually every organ system in humans and other organisms. Chronic exposure has been linked to kidney dysfunction, cardiovascular diseases, bone disorders, neurodegenerative conditions, and various forms of cancer. Essential trace elements, while necessary for normal physiological functions, can also become toxic at elevated concentrations, disrupting cellular metabolism and inducing oxidative stress. The complex interactions between toxic and essential elements further complicate our understanding of their health effects and environmental fate [14,15].

This Special Issue, entitled “Cadmium and Trace Elements Toxicity,” brings together ten high-quality research articles that advance our understanding of the sources, mechanisms, and health impacts of cadmium and trace element exposure. The contributions span multiple disciplines, from molecular toxicology and environmental monitoring to plant science and risk assessment, providing a comprehensive overview of current research in this critical field. The articles address key topics including novel toxicity mechanisms, biomonitoring approaches, protective strategies, and environmental quality criteria.

## 2. Contributions to This Special Issue

The first contribution by Schäfer et al. (Contribution 1) provides a comprehensive review of the role of trace elements in cardiovascular diseases. This article examines both essential and non-essential trace elements, emphasizing how chronic exposure to toxic metals such as cadmium, lead, and mercury can contribute to cardiovascular pathology through multiple mechanisms, including oxidative stress, inflammatory responses, and disruption of essential metal homeostasis. The review highlights the growing body of evidence linking environmental metal exposure to increased cardiovascular morbidity and mortality, underscoring the need for preventive measures and public health interventions.

Satchanska (Contribution 2) presents an extensive review of the mechanisms of cadmium toxicity in living organisms, tracing the historical recognition of cadmium as a toxicant from the mid-19th century to contemporary understanding. The article comprehensively describes cadmium’s toxicokinetics and toxicodynamics, its propensity for long-term biological retention, and its preferential accumulation in soft tissues. The review emphasizes the shift in primary cadmium pollution sources from industrial activities to phosphate fertilizers, which now represent the main contamination pathway affecting soil, water, and ultimately human health through the food chain.

Referring to gender studies, Satarug et al. (Contribution 3) demonstrate gender differences in the severity of cadmium nephropathy, examining the relationship between cadmium body burden and renal dysfunction in 448 residents from both polluted and non-polluted regions of Thailand. The study reveals that while cadmium body burden was similar between men and women, significant gender differences emerged in the severity of renal effects. The research demonstrates that cadmium-induced tubulopathy and reduced glomerular filtration rate occurred at similar exposure levels in both sexes, but the prevalence odds ratios differed, suggesting potential gender-specific susceptibility factors that warrant further investigation in risk assessment frameworks.

Building on the theme of organ-specific toxicity, Tokumoto et al. (Contribution 4) elucidate a novel mechanism based on cadmium-induced hepatic iron deficiency through a long-term animal study. Using mice chronically exposed to dietary cadmium for up to 21 months, the researchers demonstrate that cadmium causes marked decreases in liver iron concentration by suppressing the expression of iron transport-related genes in the proximal duodenum. This study reveals a previously underappreciated mechanism of cadmium toxicity, showing how chronic exposure can disrupt essential metal homeostasis and lead to secondary nutritional deficiencies, with potential implications for understanding cadmium-induced anemia and related metabolic disorders.

Forte et al. (Contribution 5) present novel findings on toxic metal and essential element concentrations in pancreatic ductal adenocarcinoma (PDAC) patients. By analyzing blood and tissue samples from PDAC patients and healthy controls using inductively coupled plasma mass spectrometry, the study reveals significantly altered levels of chromium, copper, and copper-to-zinc ratios in patient blood, as well as increased concentrations of copper, selenium, iron, and zinc in cancer tissue. These findings suggest that metal dysregulation may play a role in PDAC pathogenesis and that metal profiling could potentially serve as a diagnostic or monitoring tool for this highly aggressive malignancy.

In contrast to the human health focus of preceding articles, Vannini et al. (Contribution 6) offer an environmental perspective by comparing cadmium accumulation and release dynamics in the lichen *Evernia prunastri* and wood-derived biochar, exploring the potential use of biochar as an environmental biomonitor. The controlled laboratory experiments demonstrate that the lichen exhibits superior cadmium adsorption capacity (46.5% higher than biochar samples) but releases only about 6% of the accumulated metal. The study identifies surface area and cation exchange capacity as key determinants of cadmium sequestration ability, suggesting that biochar could serve as an alternative or complementary tool for monitoring atmospheric cadmium deposition and potentially water body contamination.

Yang et al. (Contribution 7) develop hardness-dependent freshwater quality criteria for cadmium protection of aquatic organisms in China. Based on a comprehensive toxicity database comprising 249 acute and 62 chronic toxicity data points across 52 species, the study establishes region-specific water quality criteria that account for the significant influence of water hardness on cadmium toxicity. The research identifies the most sensitive species for both short-term and long-term exposure scenarios and provides critical tools for environmental regulators to protect aquatic ecosystems from cadmium pollution while considering local environmental conditions.

Using a seed priming approach, Mudassar et al. (Contribution 8) investigate the protective effects of triacontanol against cadmium toxicity in mung bean (*Vigna radiata* L.) and demonstrate that triacontanol treatment significantly improves plant growth, photosynthetic performance, nutrient uptake, and stress tolerance under cadmium stress conditions. Notably, the optimal concentration of triacontanol (20 µM) reduced cadmium accumulation in plants by 3-fold while increasing the metal tolerance index by 6.6-fold. These findings offer promising agricultural strategies for cultivating crops in cadmium-contaminated soils while minimizing metal uptake into the food chain.

Urbano et al. (Contribution 9) examine exposure to cadmium and other trace elements among individuals with mild cognitive impairment (MCI). This cross-sectional study of 128 MCI patients from Northern Italy measured trace element concentrations in both serum and cerebrospinal fluid, providing unique insights into the potential role of environmental chemicals in cognitive decline. The study reveals complex relationships between trace elements and biomarkers of neurodegeneration, particularly involving tau proteins and β-amyloid, suggesting that both toxic and essential trace elements at dysregulated concentrations may contribute to the pathogenesis of cognitive impairment.

Finally, Wang et al. (Contribution 10) elucidate the molecular mechanisms of cadmium-induced kidney apoptosis through the IRE1α-XBP1 signaling pathway and demonstrate the protective effects of quercetin. Using a rat model, the study shows that cadmium exposure activates endoplasmic reticulum stress through the IRE1α-XBP1 pathway, leading to renal cell apoptosis and tissue damage. Importantly, quercetin supplementation significantly attenuates these effects by inhibiting the IRE1α-XBP1 pathway, suggesting that natural flavonoid compounds may offer therapeutic potential for preventing or mitigating cadmium-induced nephrotoxicity.

## 3. Conclusions and Future Perspectives

The collection of articles in this Special Issue provides valuable insights into the diverse aspects of cadmium and trace element toxicity, from molecular mechanisms to ecosystem-level impacts.

Collectively, the studies in this Special Issue advance the field by bridging molecular mechanisms with population-level health assessments and by introducing innovative approaches for environmental monitoring and agricultural remediation.This collection highlights a paradigm shift from merely documenting exposure to understanding complex inter-element interactions and developing practical intervention strategies.

Several overarching themes emerge from these contributions:

First, the articles collectively demonstrate that cadmium toxicity operates through multiple, interconnected pathways affecting virtually all biological systems. Understanding these mechanisms at the molecular level, such as the IRE1α-XBP1 signaling pathway in kidney cells or the disruption of iron homeostasis in the liver, is crucial for developing targeted interventions and identifying vulnerable populations.

Second, the research highlights significant gaps in our understanding of inter-individual variability in susceptibility to cadmium and trace element toxicity. Gender differences, genetic factors, nutritional status, and co-exposure to other elements all appear to modulate toxic responses, suggesting the need for more personalized approaches to risk assessment and prevention.

Third, the studies emphasize the importance of developing practical tools for environmental monitoring and remediation. From establishing region-specific water quality criteria to exploring novel biomonitoring approaches using biochar, these efforts are essential for protecting both human health and ecosystem integrity.

Fourth, the identification of protective strategies, whether through natural compounds like quercetin and triacontanol or through nutritional interventions, offers hope for mitigating the health impacts of cadmium exposure, particularly in populations where contamination is difficult to avoid completely.

Looking forward, several research priorities emerge from this collection. There is a critical need for long-term epidemiological studies that can better characterize the health effects of low-level, chronic cadmium exposure across diverse populations. Mechanistic studies should continue to explore the interactions between cadmium and essential trace elements, as these relationships appear central to understanding toxic outcomes. Additionally, research into cost-effective remediation strategies and protective interventions is urgently needed, particularly for agricultural systems and populations in heavily contaminated regions.

Climate change and evolving agricultural practices may alter cadmium bioavailability and exposure patterns, necessitating ongoing vigilance and adaptive management strategies. The development of early biomarkers of cadmium-induced organ damage, as well as more sophisticated modeling approaches to predict tissue-specific accumulation and toxicity, will be crucial for improving risk assessment frameworks.

These studies should serve as catalysts for future research initiatives and proposals. For instance, the findings of Satarug et al. and Urbano et al. highlight the critical need for prospective cohort studies incorporating biomonitoring data alongside genetic and nutritional covariates to elucidate individual susceptibility to chronic low-level cadmium exposure. Similarly, the promising protective effects of quercetin (Wang et al.) and triacontanol (Mudassar et al.) warrant systematic investigation into additional natural compounds and their potential synergistic mechanisms in ameliorating cadmium-induced toxicity across diverse species and tissue types.

Furthermore, certain limitations identified in some studies may serve as valuable starting points for future research endeavors. For instance, while the biochar study by Vannini et al. presents a promising novel tool for cadmium remediation, its applicability under diverse field conditions remains to be fully validated through large-scale trials and long-term monitoring programs.

In conclusion, this Special Issue advances our understanding of cadmium and trace element toxicity while highlighting the continued challenges posed by these persistent environmental pollutants. The multidisciplinary approaches represented here, combining environmental chemistry, molecular toxicology, epidemiology, and plant science, exemplify the collaborative effort required to address this complex public health issue. We hope that these contributions will stimulate further research and inform evidence-based policies to minimize human and environmental exposure to these hazardous substances.

## Data Availability

No new data were created or analyzed in this study. Data sharing is not applicable to this article.
